# Generation of deep learning based virtual non-contrast CT using dual-layer dual-energy CT and its application to planning CT for radiotherapy

**DOI:** 10.1371/journal.pone.0316099

**Published:** 2024-12-30

**Authors:** Jungye Kim, Jimin Lee, Bitbyeol Kim, Sangwook Kim, Hyeongmin Jin, Seongmoon Jung

**Affiliations:** 1 Department of Biomedical Engineering, Korea University, Seoul, Republic of Korea; 2 Department of Nuclear Engineering, Ulsan National Institute of Science and Technology, Ulsan, Republic of Korea; 3 Graduate School of Artificial Intelligence, Ulsan National Institute of Science and Technology, Ulsan, Republic of Korea; 4 Department of Radiation Oncology, Seoul National University Hospital, Seoul, Republic of Korea; 5 Department of Medical Biophysics, University of Toronto, Toronto, Canada; 6 Division of Biomedical Metrology, Ionizing Radiation Group, Korea Research Institute of Standards and Science, Daejeon, Republic of Korea; Chung-Ang University Gwangmyeong Hospital, REPUBLIC OF KOREA

## Abstract

This paper presents a novel approach for generating virtual non-contrast planning computed tomography (VNC-pCT) images from contrast-enhanced planning CT (CE-pCT) scans using a deep learning model. Unlike previous studies, which often lacked sufficient data pairs of contrast-enhanced and non-contrast CT images, we trained our model on dual-energy CT (DECT) images, using virtual non-contrast CT (VNC CT) images as outputs instead of true non-contrast CT images. We used a deterministic method to convert CE-pCT images into pseudo DECT images for model application. Model training and evaluation were conducted on 45 patients. The performance of our model, ’VNC-Net’, was evaluated using various metrics, demonstrating high scores for quantitative performance. Moreover, our model accurately replicated target VNC CT images, showing close correspondence in CT numbers. The versatility of our model was further demonstrated by applying it to pseudo VNC DECT generation, followed by conversion to VNC-pCT. CE-pCT images of ten liver cancer patients and ten left-sided breast cancer patients were used. A quantitative comparison with true non-contrast planning CT (TNC-pCT) images validated the accuracy of the generated VNC-pCT images. Furthermore, dose calculations on CE-pCT and VNC-pCT images from patients undergoing volumetric modulated arc therapy for liver and breast cancer treatment showed the clinical relevance of our approach. Despite the model’s overall good performance, limitations remained, particularly in maintaining CT numbers of bone and soft tissue less influenced by contrast agent. Future research should address these challenges to further improve the model’s accuracy and applicability in radiotherapy planning. Overall, our study highlights the potential of deep learning models to improve imaging protocols and accuracy in radiotherapy planning.

## Introduction

Dual-energy computed tomography (DECT) utilizes two X-ray tubes operating simultaneously at distinct energy levels, rapid peak kilovoltage switching, or a dual-layer detector system [1–3]. DECT offers the advantage of acquiring various parametric maps by employing pairs of high-energy and low-energy signals. The varying attenuation levels of tissues at different energy levels further enable tissue differentiation and characterization [4, 5]. However, in contrast to the affordable and widely accessible single-energy computed tomography (SECT), DECT equipment is less accessible due to its high cost. Therefore, if the parametric information of DECT images could be predicted using SECT scans, it would offer a more cost-effective approach.

One of the parametric maps obtainable from DECT scans is virtual non-contrast (VNC) images. VNC images are generated by subtracting iodine maps from contrast-enhanced (CE) DECT images [6–8]. These VNC images replace the need for pre-contrast scans, substantially enhancing dose calculation accuracy in radiation therapy [9–12]. Iodine-enhanced images exhibit increased X-ray attenuation. Yamada et al. [10] highlight that increased tissue attenuation can lead to incorrect dose calculations due to the high atomic number of iodine. In their study, VNC images achieved dose distribution pass rates over 90%, compared to 50–60% for enhanced images. Noid et al. [13] and Afifah et al. [14] also used VNC to replace the pre-contrast CT scans for radiotherapy (RT) treatment planning, eliminating registration errors between CE CT and pre-contrast CT (or cone-beam CT). However, most of the studies which dealt with X-ray RT (e.g., 3-dimensional conformal radiation therapy (3D CRT), volumetric modulated arc therapy (VMAT), and stereotactic body radiation therapy (SBRT)) have commonly concluded that the influence of contrast agents on dose calculation was not significant and clinically tolerable [15–20].

On the other hand, it was reported that heavy ion beams resulted in a 1.24 mm range difference due to the iodine contrast agent for a 5 cm tumor [21]. Furthermore, the CT number for CE CT ranged from 110 to 250 HU in the regions of the heart and great vessels, which induced a mean range error of 1.0 cm for proton radiotherapy [22]. Another study showed that the range difference decreased to 1.0 mm between TNC CT and VNC CT, whereas it was 3.2 mm between TNC CT and CE CT [23].

Liugang et al. [24] first proposed the generation of VNC images from CE CT images based on U-Net. They trained the model using two separate scans of CE CT and TNC CT. Furthermore, they calculated the dose distribution for esophageal cancer RT between CE CT and VNC CT using a commercial treatment planning system (TPS). Compared to the dose distribution calculated on TNC CT, the average gamma passing rate with a criterion of 2%/2 mm for the VNC CT image was higher than that of the CE image (99.6% and 97.3%, respectively with a p-value < 0.05).

Sui et al. [25] also generated VNC CT images from CE CT images using a U-Net. They used CE CT as the input of the model and TNC CT as the target, similar to the study conducted by Liugang et al. [24]. They acquired TNC CT and CE CT images sequentially, less than 3 minutes apart. The proton dose based on CE CT resulted in a significant difference compared to the dose calculated on TNC CT images. However, the dose calculated based on the generated VNC CT images was consistent with that based on TNC CT images.

In contrast to the aforementioned studies, we trained a model using CE CT images as inputs and VNC CT images as outputs. Both images were acquired using dual-layer DECT (DL-DECT). The VNC CT images were used in training as an alternative to TNC CT images. In our institution, practical imaging protocols in both the radiation oncology and radiology departments do not include a double scanning process (i.e., pre-contrast and post-contrast CT scans). Therefore, we were unable to obtain a sufficient number of data pairs of CE CT and TNC CT images. Using the VNC CT images as outputs for training the model may raise concerns about how accurate they are compared to TNC CT images. Several reports have shown that DECT VNC images can sufficiently subtract iodine, and therefore can be a valid alternative to TNC CT [26, 27]. Holz et al. concluded that the overall accuracy of VNC images is high and independent of dose, kernel, and denoising settings [26]. Also, VNC CT images have advantages over TNC CT images in that they are acquired simultaneously during the CT scan. In other words, there is no temporal difference between the input and output images.

Jakubicek et al. [28] presented a paired supervised image-to-image translation model-based approach for predicting TNC CT images from CE SECT images. The results qualitatively resembled the original VNC images, but quantitatively, the trained convolutional neural networks (CNNs) exhibited slight density reductions in soft tissues. Zhao et al. [29] developed a deep learning-based approach for performing DECT imaging using standard SECT data. They employed a two-step CNN to map low-energy images to high-energy images. The aortic iodine quantification difference between the original DECT and deep learning DECT images was predicted to be 0.9%, indicating high consistency with the original high-energy CT images.

The aforementioned studies utilized (i) a SECT scanner for training the model and applied it to images from the same CT scanner [24, 25] or (ii) a DECT scanner for training the model and applied it to images from the same DECT scanner [26]. However, in this study, we generated a U-Net based deep learning model using dual-layer DECT images and applied it to generate VNC images for planning CT (pCT). After generating the deep learning model using pairs of image sets from DL-DECT, the model was used to convert contrast-enhanced planning CT (CE-pCT) to VNC planning CT (VNC-pCT). To apply the model to CE-pCT images, CE-pCT images were converted from pCT to DECT images using a deterministic method. Hereafter, these deterministically generated images will be referred to as ’pseudo CE DECT’ images. Our model was developed by training on a paired dataset of DECT images and their corresponding VNC images. As a result, when the input image closely resembles a DECT image, the output image will closely match the corresponding VNC image. To validate the generated VNC-pCT images, they were quantitatively compared with true non-contrast planning CT (TNC-pCT) images. Furthermore, we also performed dose calculations on CE-pCT and VNC-pCT images from twenty patients who underwent SBRT or VMAT, including ten liver cancer patients and ten left-sided breast cancer patients.

## Material and methods

### Ethics statement

All procedures performed in this study involving human participants were in accordance with the ethical standards of the institutional review board (IRB approval No. H-1909-103-1066) and with the 1964 Helsinki Declaration and its later amendments or comparable ethical standards. Data used in this study were accessed for research purposes on 11/05/2020. Informed written consent from the patients was waived due to the retrospective nature of our study.

#### DECT image acquisition and processing

The dataset utilized in the deep learning training and evaluation comprises images obtained retrospectively from 45 patients using a DECT scanner (IQon spectral CT, Philips, the Netherlands) in 2019. The scanner features dual-layer detector arrays that obtain low- and high-energy CT data separately at the same time. The peak voltage of the X-ray tube was 120 kVp. Pairs of low- and high-energy images were used to generate VNC images. The input image referred to as the ‘CE DECT’ image in this study is the single-energy (i.e., 120 kVp) image, generated by summing the low- and high-energy data. The field of view (FOV) of the images ranged from 230 mm to 421 mm. The CE CT scans were performed in the late arterial or portal venous phase. The contrast agent used in this study was Xenetix 350^®^ (Guerbet, France) and the concentration of iodine was 350 mg/ml. All scans were performed following the administration of 1.3–1.5 ml/kg body weight. Initially, the pixel values of the images were adjusted by adding 1024 to ensure a range of 0 to 4095. Subsequently, minimum-to-maximum normalization was performed by dividing by 4095 to rescale the values to be between 0 and 1. The 45 patients were divided into sets of 31, 9, and 5 patients for training, validation, and testing, containing 6390, 1576, and 705 axial slices, respectively. For data augmentation, center cropping, which involves cropping the central part of the images and resizing them back to the original size, was applied to 10% of the training dataset. As a result, the total number of slices used for model training increased to 7029.

#### Planning CT image acquisition

Pairs of TNC-pCT and CE-pCT from 20 patients who underwent RT for liver and left-sided breast cancer were obtained retrospectively. In our institution, CE-pCT is typically used for routine treatment planning. However, there were a few patients who underwent both pre- and post-contrast scans. TNC-pCT scans were performed with a pneumatic compression plate, followed by CE-pCT scans conducted with continuous positive airway pressure (CPAP). Among the twenty patients, ten were treated for liver cancer, and ten were treated for left-sided breast cancer. The pCT scanner was a Brilliance Big Bore (BBB) CT manufactured by Philips. The peak voltage of the X-ray tube was 120 kVp, and the FOV of the images was 700 mm. The CE-pCT scans were performed across phases, with some patients imaged during the late arterial phase and others during the portal venous phase.

#### Phantom image acquisition

**Fig 1:**
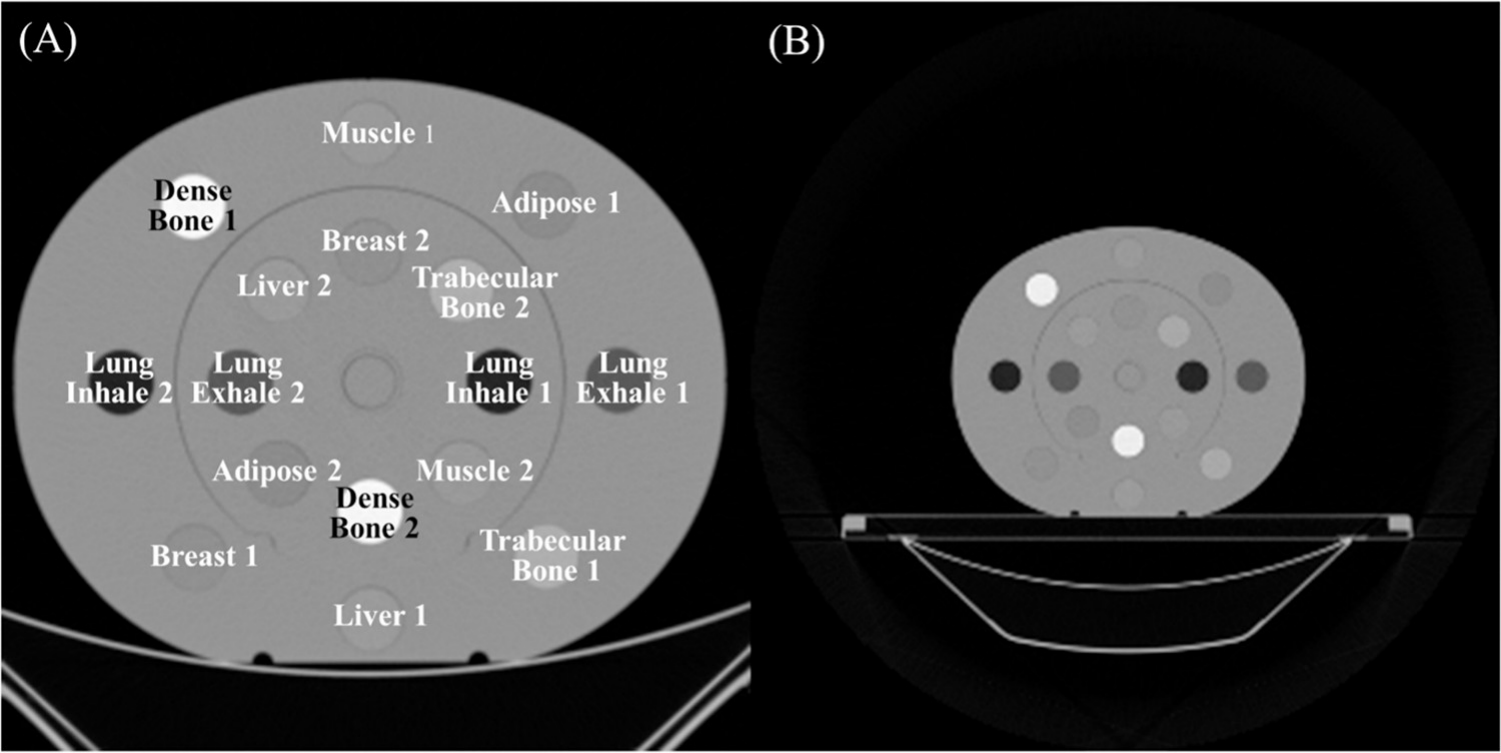
CIRS phantom CT images. The CT images of the CIRS phantom (Model 062M, Sun Nuclear Corp. FL, USA) acquired by the DECT (iQon Spectral CT, Philips, the Netherlands) (A) and planning CT scanner (Brilliance Big Bore (BBB) CT, Philips, the Netherlands) (B). CT, computed tomography; DECT, dual-energy computed tomography.

CT images of a CIRS electron density reference phantom (Model 062M, Sun Nuclear Corp. FL, USA), inserted with tissue plugs, were used to develop a deterministic pathway to generate pseudo DECT. The CT images were acquired by both the DECT scanner (iQon Spectral CT) and the pCT scanner (BBB CT). The scan protocols were the same as those used for patient imaging for each CT scanner. The information about the plugs inserted in the CIRS phantom is shown in Table 1, and the CT images acquired by the DECT and pCT scanners are shown in Fig 1. To match the FOV of DECT, resizing was performed on pCT images. After adjusting the image size, a circular ROI with a radius of 12 mm, which is 80% of the actual plug radius of 15 mm, was selected at the center of each plug in three consecutive slices to obtain the CT number. In the pCT before resizing, there were 248 pixels within the ROI; however, after resizing, there were 968 pixels within the ROI, consistent with the DECT images. The CT numbers and noise levels (given as the standard deviation) of the images from each scanner were analyzed and used to establish a relationship between the two scanners. A detailed explanation will be provided in subsequent sections. Additionally, we also acquired the DECT and pCT image sets of the CIRS phantom without dense bone plugs to assess the effect of CT number variations influenced by bone artifacts. The CT numbers and standard deviations of the tissue plugs were analyzed when all tissue plugs were present and when no dense bone plugs were present, confirming whether the presence of bone affected the CT numbers.

**Table 1:** Mass density, electron density, and relative electron density of tissue equivalent plugs inserted in CIRS electron density reference phantom (Model 062M, Sun Nuclear Corp., FL, USA).

	Mass density (g/cm^3^)	Electron density (10^23^ electrons/cm^3^)	Relative electron density^a^
Lung (inhale)	0.205	0.668	0.200
Lung (exhale)	0.507	1.658	0.496
Adipose	0.960	3.171	0.949
Breast (50% Gland / 50% Adipose)	0.990	3.261	0.976
Muscle	1.060	3.483	1.043
Liver	1.070	3.516	1.052
Trabecular bone (200mg/cc HA)	1.160	3.730	1.117
Dense bone	1.530	4.862	1.456

^a^Electron density normalized to the electron density of water.

### Model using DECT dataset

#### Model description

**Fig 2:**
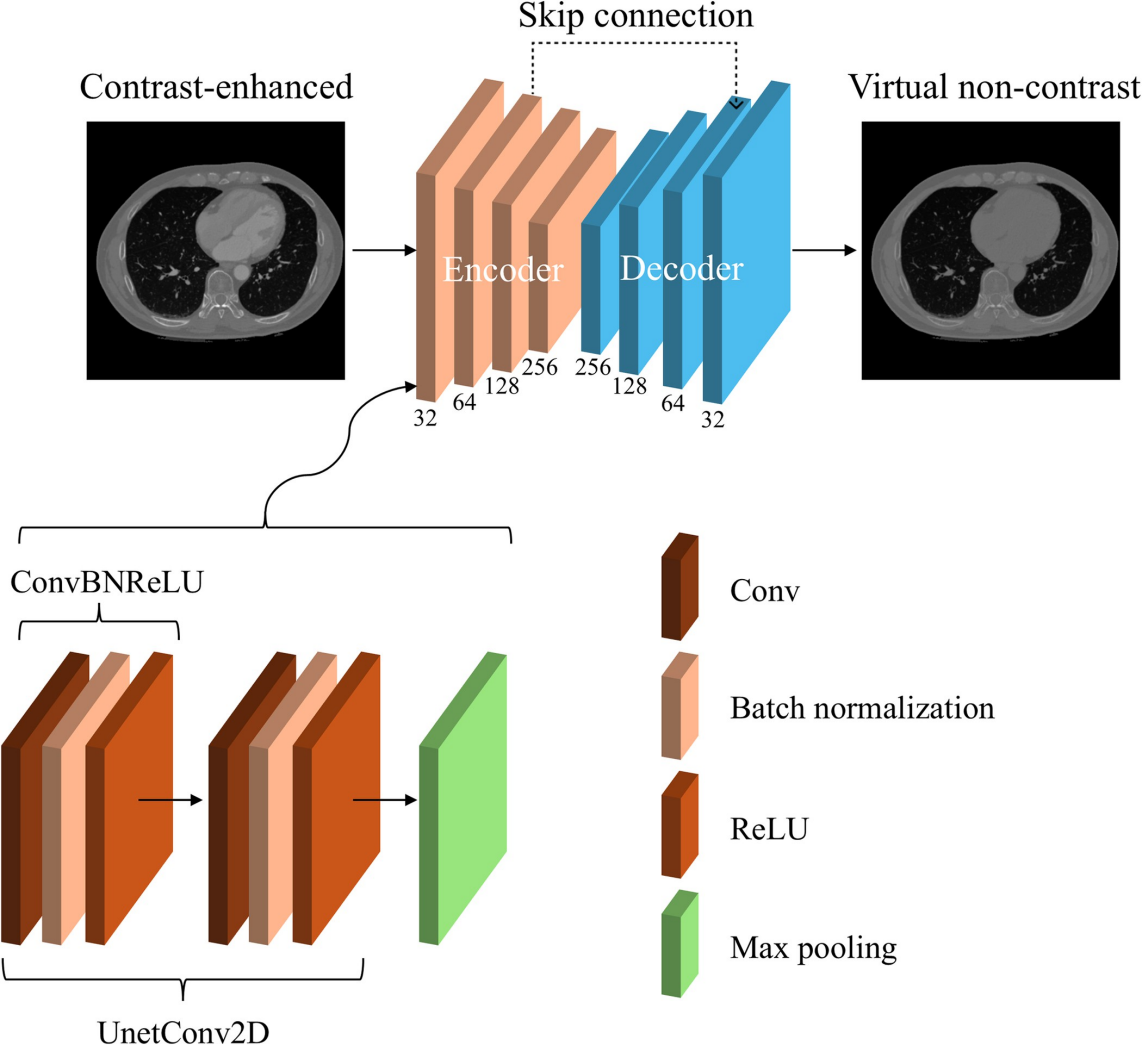
VNC-Net architecture. The model takes contrast enhanced dual-energy CT (CE DECT) images as input and generates virtual non-contrast (VNC) images as output. The numbers denoted below the encoder/decoder blocks represent the number of channels. The bottom of the figure depicts the structure of the down-sampling block of the encoder, which comprises a UnetConv2D block and a max pooling layer. The UnetConv2D component includes two ConvBNReLU blocks, each consisting of a Conv layer that executes 2D convolutional operations, followed by batch normalization and a ReLU activation function.

A deep learning model named ’VNC-Net’ is proposed to generate VNC images from CE CT scans (Fig 2). ’VNC-Net’ is based on the 2D U-Net architecture, which is widely used for tasks such as segmentation and object detection [30]. The model comprises an encoding phase that increases the number of channels while reducing dimensions to capture features of input images. Additionally, it includes a decoding phase that reduces the number of channels while increasing dimensions to reconstruct high-dimensional images. Similar to U-Net, concatenated skip connections are utilized to minimize information loss by merging features obtained from each layer of the contracting (i.e., down-sampling) path into corresponding layers of the expanding (i.e., up-sampling) path [31].

More specifically, the encoder consists of four down-sampling blocks with channel sizes of 32, 64, 128, and 256, respectively. Each block consists of a UnetConv2D block and max-pooling [32]. Max pooling is a down-sampling operation commonly used in CNNs to reduce the spatial dimensions of the input, helping to reduce computational complexity and focus on the important features. The UnetConv2D block defines a basic convolutional block, which consists of two consecutive ConvBNReLU blocks. In the ConvBNReLU block, there is a 2D convolutional layer that applies sliding convolutional filters to the input, capturing the spatial hierarchies of data features followed by batch normalization [33] and a Rectified Linear Unit (ReLU) [34] activation function. Here, batch normalization is a technique used to improve the training and performance of neural networks by normalizing the input of each layer in a mini-batch. ReLU is an activation function that introduces non-linearity to the network. For the center block, spatial information is compressed into a bottleneck feature map using a UnetConv2D block. For the decoder, there are four up-sampling blocks with channel sizes of 256, 128, 64, and 32, which symmetrically match the channel sizes of the down-sampling blocks. Each block consists of a transposed convolution operation [35], followed by concatenation with the corresponding encoder block’s output (skip connection) and a UnetConv2D block. Lastly, a final convolution, a 1×1 convolutional layer, is used to produce the final output.

#### Implementation details

We used mean squared error (MSE) as the loss function and optimized it with the Adam optimizer. Additionally, when training VNC-Net, we used a batch size of 20 and a learning rate of 0.0001 for 300 epochs. Our model was implemented using the widely used deep learning framework, PyTorch.

#### Evaluation metrics

To assess the similarity between the VNC images generated by the model and the ground truth VNC images, several evaluation metrics were employed in this study: MSE, peak signal-to-noise ratio (PSNR), structural similarity index method (SSIM), and Pearson correlation coefficient (PCC). We used the body contour masks to evaluate only the interior of the patient’s body.

MSE calculates the average squared distance between corresponding pixels of the actual and predicted images [36–38]. Let *x* and *y* be two images. The pixels of *x* are denoted as *x*_1_, *x*_2_, *x*_3_,…,*x*_*p*_, and the pixels of *y* are denoted as *y*_1_, *y*_2_, *y*_3_,…,*y*_*p*_. The MSE value between *x* and *y* is calculated according to Eq (1); the closer the value is to zero, the smaller the difference between the two images. In other words, MSE is a pixel-based error measurement.


$$MSE(x,y)=\frac{1}{p}\sum_{i=1}^{p}{\left(y_{i}-x_{i}\right)}^{2}$$ (1)

PSNR is the ratio between the maximum possible power of the signal and the power of noise, with higher values indicating better image quality [38, 39]. As shown in Eq (2), MSE is in the denominator. Therefore, as the MSE value approaches zero, the PSNR value approaches infinity. Consequently, the higher the PSNR value, the higher the image quality. Here, *peakval* is the maximal value in the data.


$$PSNR=10\frac{log(peakval^{2})}{MSE}$$ (2)

SSIM evaluates similarity to the original image [39, 40]. This metric overcomes the limitation that PSNR does not adequately reflect human perception well and aims to enhance the effectiveness of image quality assessment in the human visual system. In SSIM, the error consists of three components: luminance error (I(*x*, *y*)), contrast error (C(*x*, *y*)), and structure error (S(*x*, *y*)). When these three components are combined, the equation is given by Eq (3), where μ and σ denote the mean and standard deviation of the whole image, respectively, and ε_1_, ε_2_ (≪1) are two small positive constants [40]. SSIM was calculated using the scikit-image library, where ε_1_ and ε_2_ were automatically determined according to the image value range.


$$SSIM(x,y)=I(x,y)C(x,y)S(x,y)=\left[\frac{2\mu_{x}\mu_{y}+\varepsilon_{1}}{\mu_{x}^{2}+\mu_{y}^{2}+\varepsilon_{1}}\right]\left[\frac{2\sigma_{xy}+\varepsilon_{2}}{\sigma_{x}^{2}+\sigma_{y}^{2}+\varepsilon_{2}}\right].$$ (3)

PCC is a metric that quantifies the linear correlation between two variables *x* and *y* [41, 42]. When *σ*_*x*_ and *σ*_*y*_ are the standard deviations of the pixels in images *x* and *y*, and *σ*_*xy*_ is the covariance between *x* and *y*, PCC is expressed as shown in Eq (4). It ranges from -1 to +1, with +1 indicating a perfect positive linear correlation, -1 indicating a perfect negative linear correlation, and 0 indicating no relationship between the two variables. Therefore, in image quality assessment, a PCC value closer to +1 indicates that the generated image is more consistent with the actual image.


$$\rho (x,y)=\frac{\sigma_{xy}}{\sigma_{x}\sigma_{y}}$$ (4)

#### Comparison between CE DECT, VNC DECT, and VNC-Net image

The CT number analysis was performed by selecting regions of interest (ROIs). For four of the five patients in the test set, ROI analysis included the right atrium, descending aorta, and rib, while for three of these patients, it also encompassed the liver and kidney. The average CT number was calculated from the ROI volume of three consecutive slices. One of the five test set patients was excluded from the ROI analysis because it was a head and neck cancer patient, and therefore did not have the same regions as the other four patients.

### Generation of pseudo DECT and inverting process

#### Conversion of planning CT to pseudo DECT

One major concern regarding the conversion of pCT to pseudo DECT is the different sizes of FOV. The pixel size of the pCT was 1.3672 mm x 1.3672 mm with a 700 mm diameter FOV. The pixel size was reduced to 0.68 mm x 0.68 mm and the body was cropped to fit the FOV of 350 mm for the DECT. Therefore, a certain part of the body for several patients (i.e., the arms of left-sided breast cancer patients) was not converted to pseudo DECT. A bilateral filter was applied to this resized pCT using the MATLAB R2019a function *imbilatfilt* (The MathWorks Inc., Massachusetts, USA) to create a de-noised pCT. In this case, a degree of smoothing value of 109.2218, obtained from the uniform regions within the phantom, was used. The spatial sigma parameter, which determines the filter’s spatial extent, was set to 2 to enhance spatial smoothing.

Subsequently, a CT number calibration process was performed. CT imaging and analysis of the CIRS phantom were conducted to obtain the necessary information for the conversion process between pCT and DECT. Using the average CT numbers of the same tissue plugs from pCT and DECT, two piecewise linear functions were fitted with 0 HU as the boundary. Using these linear relationship functions, the pCT images were converted to pseudo DECT images. The standard deviations of the pixel values in the tissue plugs, indicating the noise level of the CT images from the two CT scanners, were also analyzed. Lastly, white Gaussian noise was added to the CT number calibration-applied image to mimic the noise level of DECT. In the Gaussian filter, the desired signal-to-noise ratio was set to 40 dB. This value was measured by randomly selecting a rectangular region within the phantom that contained no plugs and maintained a uniform pixel intensity. The average energy (i.e., signal power) of the input signal was automatically calculated, and noise was added based on the specified signal-to-noise ratio using the MATLAB R2019a function *awgn*. The standard deviation of the pixel values of the CIRS phantom, which represents the noise level, was utilized. Since the standard deviation in DECT images tends to be slightly lower than in pCT images, noise was added to match the DECT noise level. Through these processes, the pCT was converted into the final pseudo DECT.

During the transformation process, the couch was removed by first creating a binary image using a threshold value, in this case, -430 HU, to make the lung regions empty. Next, connected components were identified, and areas connected by pixels with a value below a certain threshold (7000 pixels in this case) were removed. By filling the empty spaces between pixels, specifically the empty lung regions, the result is an image where only the body interior is represented as 1 and the exterior as 0. This image is then multiplied with the pCT, effectively removing the couch.

#### Generation of pseudo VNC DECT and inversion to VNC planning CT

After the generation of the pseudo DECT, the deep learning model was applied to the pseudo DECT to generate pseudo VNC DECT. Subsequently, the pseudo VNC DECT was inverted back to the pCT for implementation in TPS dose calculation. The inversion process is the inverse of the conversion process described in the previous section. The pixel size of the cropped image was increased to 1.3672 mm × 1.3672 mm and the converted image was reinserted into the original CE-pCT. In this manner, the areas that were removed in the previous step (e.g., the arms) remained unchanged in the final VNC planning CT image.

#### Comparison between VNC planning CT and true non-contrast planning CT

The images of VNC-pCT were compared with the TNC-pCT and the CE-pCT images. The comparison between VNC-pCT and CE-pCT demonstrates how effectively VNC-Net can eliminate contrast regions. The comparison between VNC-pCT and TNC-pCT presents the accuracy of VNC-pCT. The conditions of patients during the acquisition of TNC-pCT and CE-pCT scanning differ due to the use of CPAP for CE-pCT, a compression belt for TNC-pCT, and temporal differences. An inherent limitation exists for analyzing the accuracy of VNC-pCT due to the limited dataset in this study. However, we attempted to compare the ROIs in the same position for TNC-pCT and VNC-pCT with the assistance of trained radiation oncologists. The ROIs were in the right atrium, descending aorta, lung, breast, liver, kidney, and rib.

### Treatment planning

#### Treatment plan and plan evaluation

The VMAT or SBRT plan for ten liver cancer patients and the VMAT plan for ten left-sided breast cancer patients were used. The prescribed doses and fractions for the ten liver cancer plans were 5 Gy × 10 fractions (4 plans), 7.5 Gy × 8 fractions (1 plan), 10 Gy × 5 fractions (1 plan), 12.5 Gy × 4 fractions (2 plans), and 15 Gy × 4 fractions (2 plans). The original plans for liver cancer were generated on 4D CE-pCT with average intensity projection using Varian Eclipse 16.1 TPS (Varian–a Siemens Healthineers company, Palo Alto, CA, USA). The beams used were 6 MV flattening filter free beams of TrueBeamSTx (Varian–a Siemens Healthineers company, Palo Alto, CA, USA) or Halcyon (Varian–a Siemens Healthineers company, Palo Alto, CA, USA). The treatment plans for the liver cancer patients involved two 224° partial arcs (181°–45° clockwise and 181°–45° counterclockwise) for six plans, two 219° partial arcs (181°–40° clockwise and 181°–40° counterclockwise) for two plans, two 239° partial arcs (181°–60° clockwise and 181°–60° in counterclockwise) for one plan, and two full arcs for one plan. The prescribed dose and fraction for the ten left-sided breast patients were 2.7 Gy × 16 fractions. The original plans were generated on 3D CE-pCT with 6 MV X-ray beam of VitalBeam (Varian–a Siemens Healthineers company, Palo Alto, CA, USA) using the same TPS. The ten left-sided breast plans consisted of two or three 220° partial arcs (300°–160° clockwise and 160°–300° counterclockwise). For the three partial arcs, an additional arc of 300°–160° clockwise was used. In this study, only the 50% phase CT from the 4D CT dataset was extracted for the liver cancer patients. The planning target volume (PTV) and organ at risks (OARs) were contoured on the 50% phase CE-pCT, and the dose distribution was recalculated using the original plan. The plan was recalculated on the VNC-pCTs, applying the same plan parameters as those used for the original plan.

The dosimetric differences between the plans on CE-pCT and VNC-pCT were evaluated using dose-volume histogram (DVH) parameters. The mean dose (D_mean_) of the PTV, as well as the near-maximum, median, and near-minimum doses (given by D_2%_, D_50%_, D_98%_) to the PTV, and the homogeneous index (HI) were assessed. The D_mean_ of the liver, along with the volumes of the liver receiving 20 Gy, 10 Gy, 5 Gy (V_20Gy_, V_10Gy_, V_5Gy_), were compared for liver cancer patients. The D_mean_, V_20Gy_, V_10Gy_, and V_5Gy_ of the left lung, right lung, both lungs, and heart were evaluated for left-sided breast cancer patients [43]. In addition, 2D dose difference and 2D global gamma analyses were performed with a criterion of 2%/2 mm and a low dose threshold of 10% of the maximum dose. The 2D dose plane showing the PTVs and contrast-enhanced tissues in the CE-pCT slice was selected.

### Schematic overview

**Fig 3:**
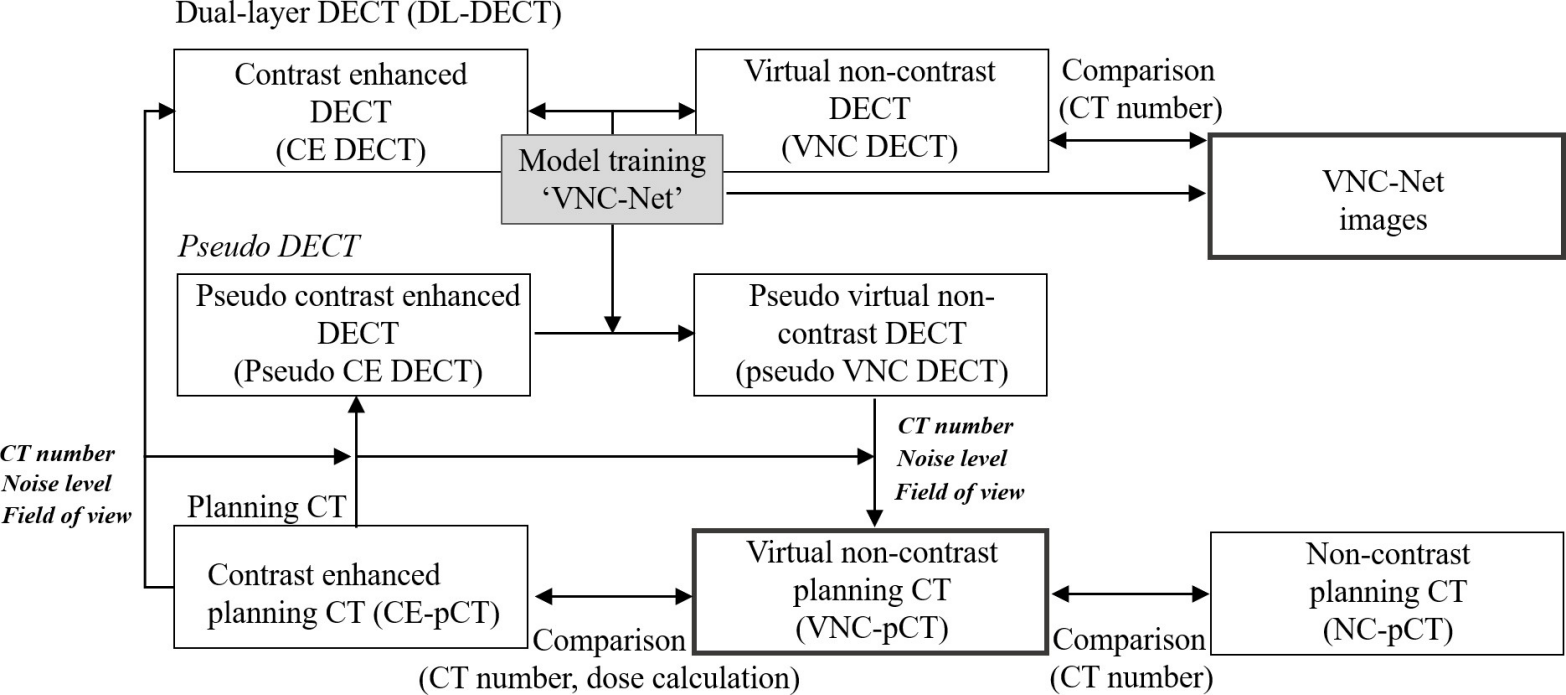
Proposed method’s pipeline. This is the tree diagram of the proposed method’s pipeline.

The whole pipeline is visualized in Fig 3 and explained in detail in the above sections.

## Results

### VNC-net model using DECT dataset

#### Evaluation metric

The model achieved results of 0.9997697, 0.9995764, 53.2774759, and 0.0000076 for the PCC, SSIM, PSNR, and MSE, respectively. The averages were calculated by aggregating the data from the test patients, indicating that the model demonstrated quantitatively good results.

#### Quantitative evaluation for regions of interest for VNC-Net

**Fig 4:**
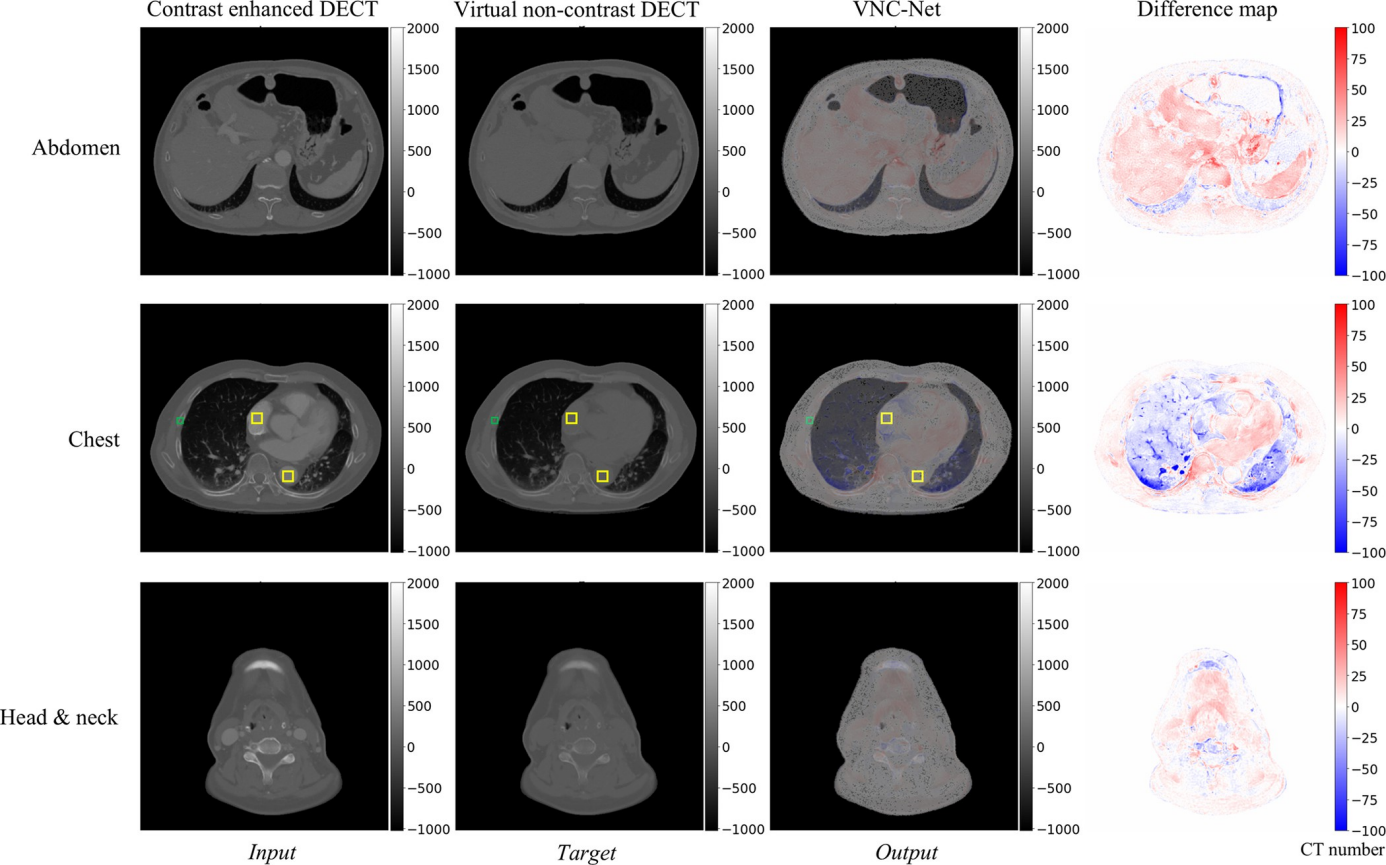
VNC-Net model results for DECT dataset. Three samples from the test set are visualized. The rightmost column represents the difference map between the target and model output, and it is overlaid on the model output in the third column to highlight where the anatomy differs from the target. The images in the middle row have the right atrium (marked by the upper left yellow box of each image), the descending aorta (marked by the lower right yellow box of each image), and the rib (marked by the green box of each image) as the ROIs. DECT, dual-energy computed tomography; ROI, region of interest.

Overall, the CT numbers for VNC-Net images and the VNC DECT images were similar as shown in Table 2. The CT number differences between the VNC DECT images and the VNC-Net images were -3 HU, 2 HU, 16 HU, -2 HU, and -3 HU in the right atrium, descending aorta, liver, kidney, and rib, respectively. In the generated VNC-Net images, the CT numbers corresponding to the contrast regions of the input image decreased similarly to those of the VNC DECT images. Furthermore, as can be seen in Fig 4, structures such as organs and bones are depicted sharply without distortion or artifacts. This indicates that the model is well-trained to eliminate contrast and can successfully generate VNC images from the input contrast images. The CT number differences between the ROIs of the input (CE DECT), target (VNC DECT), and output (VNC-Net) images generated by the model were also calculated. The ROIs were indicated by the yellow boxes, as shown in the middle row of Fig 4. The differences between CE DECT and VNC DECT were 212 HU, 209 HU, 39 HU, 115 HU, and 111 HU, respectively, while those between CE DECT and VNC-Net were 209 HU, 211 HU, 55 HU, 113 HU, and 108 HU in the right atrium, descending aorta, liver, kidney and rib, respectively.

**Table 2:** CT numbers (HU) of ROIs in the images of test dataset. CE DECT (denoted as (A)), VNC DECT (denoted as (B)), and VNC-Net (denoted as (C)) were analyzed. Numbers in parentheses are standard deviations. Statistical significance (p-value < 0.05) was assessed by Wilcoxon signed rank test. CE DECT, contrast enhanced dual-energy computed tomography; CT, computed tomography; HU, Hounsfield unit; ROI, region of interest; VNC DECT, virtual non-contrast dual-energy computed tomography.

	CE DECT DECT (A)	VNC DECT (B)	VNC-Net (C)	p-value between (A) and (B)	p-value between (A) and (C)	p-value between (B) and (C)
Right atrium (N = 4)	248 (143)	36 (5)	39 (9)	0.125	0.125	0.625
Descending aorta (N = 4)	251 (102)	42 (6)	40 (6)	0.125	0.125	0.825
Liver (N = 3)	99 (18)	60 (7)	44 (1)	0.250	0.250	0.250
Kidney (N = 3)	150 (9)	35 (4)	37 (1)	0.250	0.250	0.750
Rib (N = 4)	201 (61)	90 (37)	93 (42)	0.125	0.125	0.625

An inherent limitation of VNC can be observed in the reduction of CT numbers in the bone area (i.e., rib in Fig 4 and Table 2), which should remain consistent between the CE DECT and VNC DECT. The decrease in CT numbers for the bone region in VNC-Net images, which should not be influenced by the presence of contrast, is attributed to the lower CT numbers assigned to the bone area in the VNC DECT images compared to the input images. In other words, this suggests that as the model learns from such input-target pairs during training, it adapts not only to removing the contrast from the target VNC but also to the reduced CT numbers in the bone regions of the target VNC image. Therefore, this can be considered a flaw of the model trained on VNC images rather than TNC images.

### Generation of pseudo DECT from planning CT

#### Determination of CT number conversion

**Fig 5:**
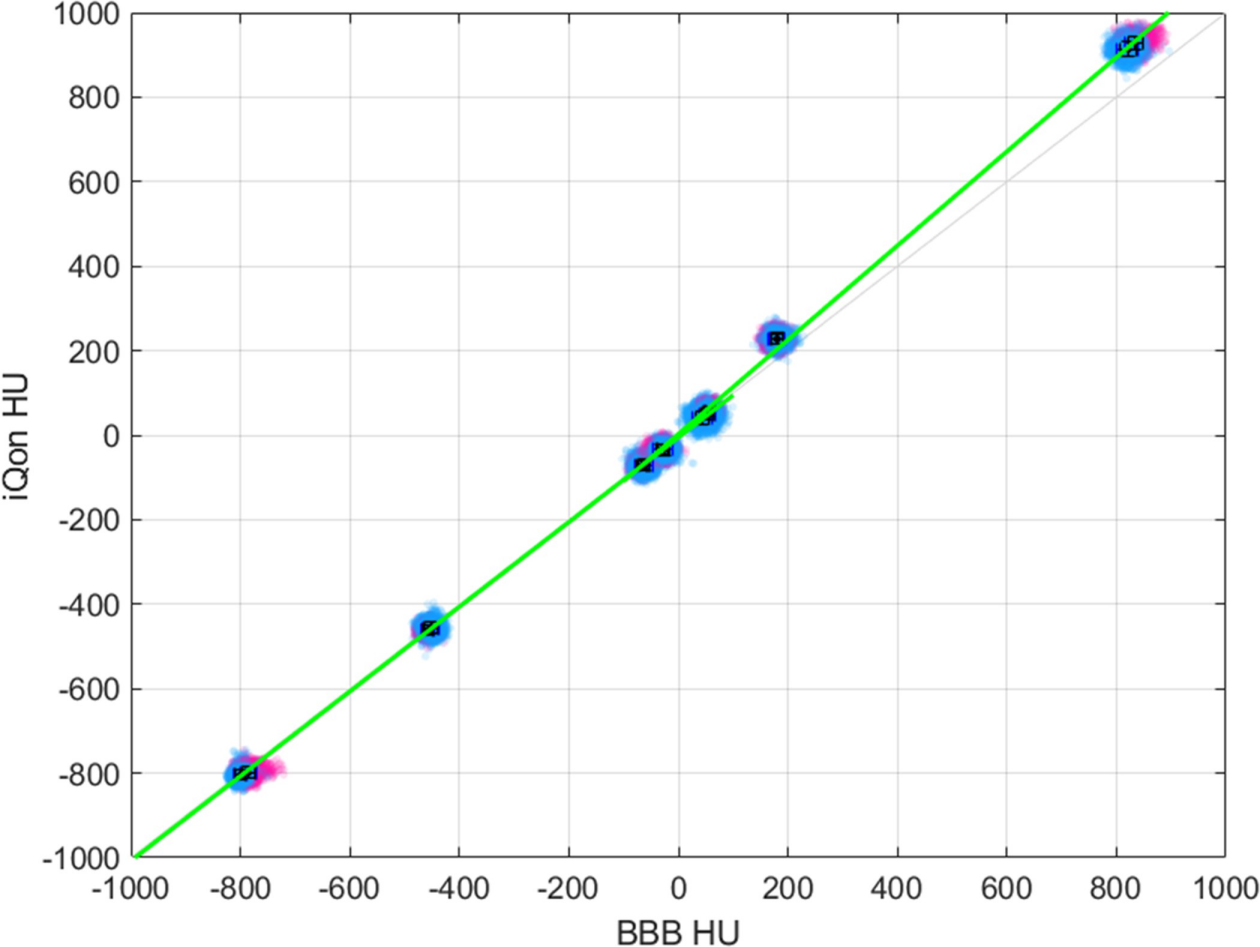
Linear relationship between the CT numbers from pCT and DECT. The linear relationship obtained from the pixel values of the pCT (Brilliance Big Bore (BBB) CT, Philips, the Netherlands) and DECT (iQon Spectral CT, Philips, the Netherlands) images of the CIRS phantom (Model 062M, Sun Nuclear Corp., FL, USA) with all tissue plugs. We obtained two linear equations for values below or equal to 0 HU and above 0 HU. Magenta and blue represent pixel values from the same tissue plug at different locations, while black represents the mean value plotted, with error bars indicating the standard deviation. The error bars are not clearly discerned from the data points due to their small standard deviations. DECT, dual-energy computed tomography; HU, Hounsfield unit; pCT, planning computed tomography.

We obtained CT numbers and standard deviations for tissue plugs to facilitate the conversion process between pCT and DECT (Table 3). It was found that the CT numbers and standard deviations did not significantly differ with or without the bone plugs. When averaging the CT numbers for two plugs in each type of tissue, the difference between the CIRS phantom with all tissue plugs and the CIRS phantom without dense bone plugs was within 3 HU. Therefore, we concluded that the bone artifact did not significantly influence the CT numbers. Using the linear relationship derived from the tissue plug data, we performed CT number conversion using Eq (5), as depicted in Fig 5. For the same plug imaged in each CT, the average value of the pCT and DECT corresponds to x and y in Eq (5), which is a piecewise linear function of the CT numbers of the pCT, with sections for values less than or equal to 0 HU and values greater than 0 HU, respectively. In other words, the horizontal axis in the graph of Fig 5 represents x, while the vertical axis represents y, and two separate linear fittings were performed using 0 HU as the boundary. Fig 5 depicts the pixel values of each plug (968 pixels in each plug) plotted as points. We performed mean CT number transformation using linear fitting equations, and R^2^ values were 0.9999 for values less than or equal to 0 HU and 0.9983 for values greater than 0 HU, respectively.


$$y=\left\{ \begin{array}{c} 1.0021x-5.3595,\;x\leq 0\\ 1.1148x+2.7053,\;x>0 \end{array}\right.$$ (5)

**Table 3:** CT numbers (HU) of a CIRS phantom (Model 062M, Sun Nuclear Corp., FL, USA) with all tissue plugs and CIRS phantom without dense bone plugs. Numbers in parentheses are standard deviations. CT, computed tomography; DECT, dual-energy computed tomography; HU, Hounsfield unit; pCT, planning CT.

	CIRS phantom with all tissue plugs			CIRS phantom without dense bone plugs		
Tissue plug	pCT	DECT	Pseudo DECT	pCT	DECT	Pseudo DECT
Lung (Inhale) 1	-785 (15)	-798 (13)	-792 (13)	-793 (15)	-808 (12)	-800 (13)
Lung (Inhale) 2	-799 (12)	-804 (13)	-807 (10)	-785 (11)	-800 (12)	-791 (10)
Lung (Exhale) 1	-457 (12)	-461 (10)	-464 (9)	-453 (11)	-460 (11)0	-459 (9)
Lung (Exhale) 2	-452 (15)	-457 (14)	-458 (11)	-454 (11)	-461 (11)	-460 (10)
Adipose 1	-67 (12)	-70 (11)	-73 (10)	-65 (9)	-70 (10)	-71 (9)
Adipose 2	-63 (15)	-70 (14)	-68 (12)	-66 (10)	-71 (10)	-72 (9)
Breast 1	-31 (15)	-34 (12)	-36 (11)	-29 (9)	-33 (10)	-35 (9)
Breast 2	-26 (14)	-35 (13)	-31 (11)	-26 (12)	-34 (12)	-32 (9)
Muscle 1	48 (11)	47 (10)	56 (9)	48 (9)	47 (9)	56 (10)
Muscle 2	44 (17)	41 (15)	52 (14)	48 (13)	45 (13)	55 (11)
Liver 1	57 (11)	56 (11)	66 (10)	56 (10)	54 (10)	64 (10)
Liver 2	53 (15)	50 (15)	62 (13)	57 (13)	51 (13)	65 (11)
Trabecular bone 1	179 (14)	228 (12)	203 (12)	178 (11)	232 (12)	201 (9)
Trabecular bone 2	180 (14)	228 (14)	204 (13)	177 (13)	229 (14)	200 (10)
Dense bone 1	833 (17)	929 (15)	933 (17)			
Dense bone 2	820 (20)	913 (17)	917 (17)			

#### Pseudo DECT and virtual non-contrast planning CT

**Fig 6:**
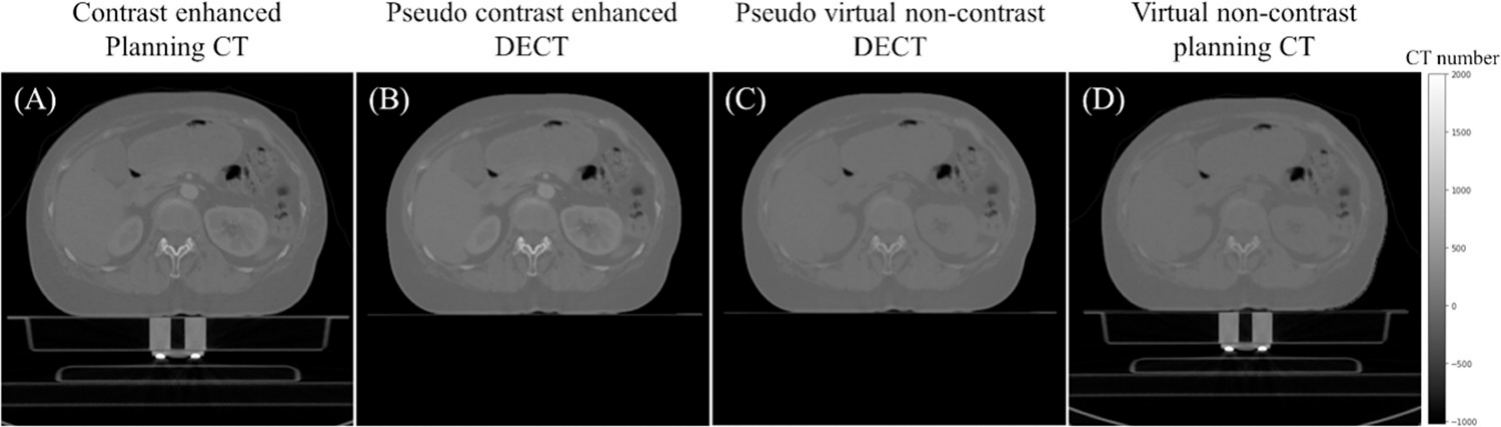
Conversion between pseudo DECT and pCT. CE-pCT (A), pseudo DECT (B), pseudo VNC DECT (C), and VNC-pCT (D). To ensure the anatomy is clearly visible, CE-pCT and VNC-pCT were enlarged to match the body size of pseudo DECT and pseudo VNC DECT. CE-pCT, contrast enhanced planning computed tomography; DECT, dual-energy computed tomography; pCT, planning CT; VNC DECT, virtual non-contrast dual-energy CT; VNC-pCT, virtual non-contrast planning computed tomography.

The standard deviations of the CIRS phantom plugs’ CT numbers were slightly higher in pCT images compared to DECT images (as shown in Table 3). We adjusted the noise level accordingly for the pseudo DECT, resulting in a similar level of standard deviation. The pseudo DECT, converted from the CE-pCT, was then fed into our proposed trained model. VNC-Net and pseudo VNC DECT were subsequently generated (as shown in Fig 6(B) and 6(C)). Fig 6(D) shows the VNC-pCT inverted back to match the scale and quality of the planning CT.

#### Comparison between contrast enhanced planning CT, true non-contrast planning CT and virtual non-contrast planning CT

**Fig 7:**
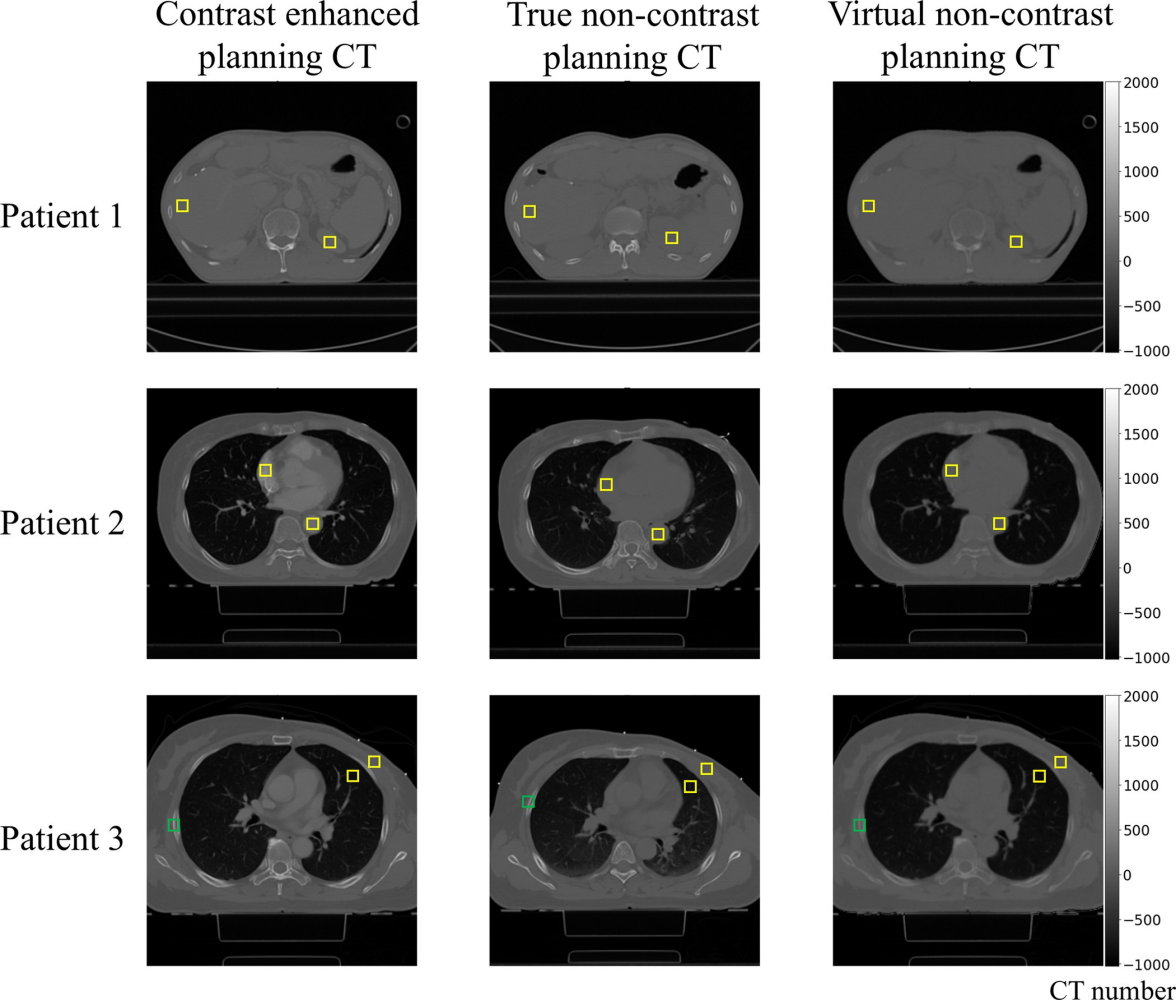
ROI analysis of the VNC-pCT results. In the top row, the ROI on the left side of each image is the liver, and the ROI on the right side is the kidney. In the middle row, the ROI in the upper left side of each image is the right atrium, and the ROI in the lower right side is the descending aorta. Lastly, in the bottom row, the ROI marked with a green box represents the ribs, while among the two ROIs marked with yellow boxes, the one on the left is the lung and the one on the right is the breast. ROI, region of interest; VNC-pCT, virtual non-contrast planning computed tomography.

An ROI analysis was conducted on the VNC-pCT results, with several cases shown in Fig 7. As presented in Table 4, for the ten breast cancer patients, the differences between TNC-pCT and VNC-pCT were 9 HU and 21 HU in the right atrium and descending aorta, respectively (all p-values < 0.05). The CT number differences between CE-pCT and TNC-pCT were 221 HU and 187 HU, respectively, while those between CE-pCT and VNC-pCT were 230 HU and 208 HU, in the right atrium and descending aorta, respectively (all p-values < 0.05). For the ROIs in lung and breast tissues, the differences between CE-pCT and TNC-pCT were -54 HU (p-value < 0.05) and -1 HU (p-value > 0.05), respectively, while those between CE-pCT and VNC-pCT were 16 HU (p-value < 0.05) and 13 HU (p-value < 0.05), respectively. The difference in lung tissue between CE-pCT and TNC-pCT was due to the use of CPAP during the CE-pCT scan. Also, the differences between TNC-pCT and VNC-pCT were 70 HU (p-value < 0.05) and 14 HU (p-value > 0.05) in the lung and breast tissues, respectively. For the ten liver cancer patients, the differences between TNC-pCT and VNC-pCT were 16 HU and 6 HU, in the liver (p-value = 0.0371) and kidney (p-value > 0.05), respectively. The differences between CE-pCT and TNC-pCT were 32 HU and 73 HU, respectively, while those between CE-pCT and VNC-pCT were 48 HU and 79 HU, in the liver and kidney, respectively (all p-values for these comparisons < 0.05). For the twenty patients, the CT number difference in the ROIs of the rib was -13 HU between CE-pCT and TNC-pCT, while it was 97 HU between CE-pCT and VNC-pCT. The difference between TNC-pCT and VNC-pCT was 110 HU (p-value < 0.001). As observed, consistent with the findings from the DECT results, that the CT numbers for the bone region decreased in the VNC-pCT compared to the input CE-pCT image.

**Table 4:** CT number (HU) of ROIs in the images of CE-pCT (denoted as (A)), TNC-pCT (denoted as (B)), and VNC-pCT (denoted as (C)) were analyzed. Statistical significance (p-value < 0.05) was assessed by Wilcoxon signed rank test. Numbers in parentheses are standard deviations. CE-pCT, contrast enhanced planning computed tomography; CT, computed tomography; HU, Hounsfield unit; ROI, region of interest; TNC-pCT, true non-contrast planning computed tomography; VNC-pCT, virtual non-contrast planning computed tomography.

	CE-pCT (A)	TNC-pCT (B)	VNC-pCT (C)	p-value between (A) and (B)	p-value between (A) and (C)	p-value between (B) and (C)
Right atrium (N = 10)	238 (122)	17 (24)	8 (16)	**0.0195**	**0.0195**	0.1933
Descending aorta (N = 10)	217 (60)	30 (13)	9 (3)	**0.0195**	**0.0195**	**0.0195**
Lung (N = 10)	-882 (37)	-828 (32)	-898 (23)	**0.0020**	**0.0020**	**0.0020**
Breast (N = 10)	-20 (62)	-19 (64)	-33 (50)	1.000	**0.0490**	0.0840
Liver (N = 10)	78 (24)	46 (22)	30 (11)	**0.0020**	**0.0020**	**0.0371**
Kidney (N = 10)	90 (34)	17 (27)	11 (15)	**0.0020**	**0.0020**	**0.0840**
Rib (N = 20)	144 (55)	157 (63)	47 (45)	**0.7285**	**< 0.001**	**< 0.001**

### Treatment planning for radiotherapy

The results of dosimetric evaluations for RT planning on CE-pCT and VNC-pCT for left-sided breast cancer patients are shown in Table 5. Since the contrast-enhanced region was neither adjacent to the PTV nor in the beam’s path, the dosimetric indices for the PTV were not significantly affected by the elimination of the contrast agent. The differences were less than 0.3%. For the OARs, a slight increase in D_mean_ (9.1 cGy_,_ 4.8 cGy, and 3.0 cGy for the left lung, right lung, and heart, respectively) and V_5 Gy_ (0.8 pp_,_ 0.6 pp, and 0.3 pp for the left lung, right lung, and heart, respectively) was observed. The relative differences were less than 0.8% and these differences were clinically tolerable.

**Table 5:** The dosimetric evaluations for treatment planning on CE-pCT and VNC-pCT for ten breast cancer patients who underwent VMAT. Numbers in parentheses are standard deviations. CE-pCT, contrast enhanced planning computed tomography; HI, homogeneity index; PTV, planning target volume; VMAT, volumetric modulated arc therapy; VNC-pCT, virtual non-contrast planning computed tomography.

	CE-pCT	VNC-pCT	p-value
Left-sided breast PTV			
D_mean_ (%)	106.2 (1.3)	106.4 (1.2)	0.1097
D_2_ (%)	110.5 (2.0)	110.7 (1.9)	0.0273
D_50_ (%)	106.8 (1.5)	107.1 (1.4)	0.0116
D_98_ (%)	98.0 (0.5)	98.0 (0.7)	0.8335
HI	0.1182 (0.0219)	0.1165 (0.0213)	0.2324
Left lung			
D_mean_ (cGy)	1132.7 (141.0)	1141.8 (138.5)	0.0371
V_20Gy_ (%)	21.1 (3.8)	21.3 (3.8)	0.0658
V_10Gy_ (%)	39.0 (5.9)	39.7 (5.7)	0.0076
V_5Gy_ (%)	58.3 (6.9)	59.1 (6.7)	0.0020
Right lung			
D_mean_ (cGy)	358.6 (45.9)	363.4 (47.1)	0.0039
V_20Gy_ (%)	0.1 (0.3)	0.2 (0.3)	0.3173
V_10Gy_ (%)	3.8 (2.9)	4.0 (3.0)	0.0421
V_5Gy_ (%)	25.0 (6.5)	25.6 (6.7)	0.0020
Both lung			
D_mean_ (cGy)	718.3 (75.1)	725.1 (74.4)	0.0195
V_20Gy_ (%)	9.9 (1.9)	10.0 (1.9)	0.1493
V_10Gy_ (%)	20.2 (2.9)	20.6 (2.9)	0.0059
V_5Gy_ (%)	40.4 (5.4)	41.1 (5.4)	0.0020
Heart			
D_mean_ (cGy)	400.8 (88.2)	403.8 (89.9)	0.0840
V_20Gy_ (%)	1.0 (0.8)	1.0 (0.8)	0.6547
V_10Gy_ (%)	7.6 (3.3)	7.7 (3.4)	0.1091
V_5Gy_ (%)	23.2 (8.3)	23.5 (8.5)	0.0644

The results of dosimetric evaluations for RT planning on CE-pCT and VNC-pCT for liver cancer patients are shown in Table 6. Since the difference in the CT numbers for the liver between CE-pCT and VNC-pCT was 48 HU, the dosimetric indices for the PTV were not significantly impacted by the reduced contrast agent. For the entire liver, an increase of 8.0 cGy in D_mean_, and 0.2–0.3 pp in V_5 Gy_, V_10 Gy_, and V_20 Gy_ were observed. For the liver PTVs, the differences were 1.1 pp, 0.9 pp, 1.0 pp, and 1.1 pp for D_mean_, D_2_, D_50_, and D_98_, respectively. The difference in HI was not statistically significant (p-value > 0.05). The relative differences were less than 1.2% and these differences were clinically tolerable for VMAT or SBRT treatment for liver cancer.

**Table 6:** The dosimetric evaluations for treatment planning on CE-pCT and VNC-pCT for ten liver cancer patients who underwent VMAT or SBRT. CE-pCT, contrast enhanced planning computed tomography; HI, homogeneity index; PTV, planning target volume; SBRT, stereotactic body radiotherapy; VMAT, volumetric modulated arc therapy; VNC-pCT, virtual non-contrast planning computed tomography.

	Contrast enhanced planning CT (CE-pCT)	Virtual non-contrast planning CT (VNC-pCT)	p-value
Liver PTV			
D_mean_ (%)	102.3 (1.7)	103.3 (1.5)	0.0020
D_2_ (%)	105.6 (2.4)	106.5 (2.1)	0.0039
D_50_ (%)	102.6 (1.9)	103.6 (1.8)	0.0020
D_98_ (%)	95.9 (6.6)	97.0 (5.8)	0.1232
HI	0.0946 (0.0679)	0.09207 (0.0638)	0.9218
Liver			
D_mean_ (cGy)	801.4 (438.2)	809.4 (441.6)	0.0073
V_20Gy_ (%)	12.7 (9.0)	12.9 (9.1)	0.0115
V_10Gy_ (%)	24.9 (12.8)	25.2 (12.8)	0.0020
V_5Gy_ (%)	37.4 (18.1)	37.6 (18.1)	0.0106

We also evaluated the 2D gamma passing rate (criterion: 2 mm/2%) between the dose distributions on CE-pCT and VNC-pCT. For the twenty patients, the minimum gamma passing rate was 99.5%, and the average gamma passing rate was 99.9%. The dose distributions on CE-pCT and VNC-pCT for VMAT and SBRT for left-sided breast cancer and liver cancer patients were identical in terms of gamma analysis.

## Discussion

### Summary

Our study employed a deep learning model, VNC-Net, trained on a DECT image dataset to generate VNC images from CE CT scans. This process involved a deterministic method to generate pseudo DECT images, ensuring consistency in pixel values, pixel size (or FOV), and noise level between the planning CT and DECT. The deep learning model was then applied to these pseudo DECT images to produce pseudo VNC DECT images, which were subsequently converted to VNC-pCT for dose calculation with the TPS. The model evaluation yielded high scores across various metrics, demonstrating fidelity in replicating target VNC images, with CT numbers showing close correspondence. The reduction of contrast was evident without causing distortion or alterations in other parts of the image. However, discrepancies in bone CT numbers between input contrast images and VNC images were noted, likely attributed to dataset characteristics.

Additionally, the application of our model to pseudo VNC DECT generation demonstrated its versatility, enabling meaningful comparisons with non-contrast images. VNC DECT images were converted back to VNC-pCT. The average CT numbers of the ROIs in the liver, kidney, heart, and great vessels of VNC-pCT images closely matched those of TNC-pCT.

### TPS plan

As presented in previous studies [10–20], CE-pCT could mostly lower the dose to targets; in other words, it could result in an overdose to TNC-pCT. However, these studies also concluded that the dose differences were clinically insignificant for cancer patients across various anatomical regions. Our study also supports those results, showing that the dose difference between CE-pCT and VNC-pCT for left-sided breast cancer undergoing VMAT technique was clinically insignificant. For the liver cancer patients, we found only small dose differences in the PTV between CE-pCT and VNC-pCT, which were clinically tolerable. While this study limits the application of VNC-pCT to VMAT or SBRT plans for X-ray RT, it has strong potential for use in proton or heavy ion beam therapy. In the previous study [21], a 57 HU difference was reported to induce a 2.5% maximum error in heavy ion range calculations. We observed that the CT number differences between CE-pCT and VNC-pCT for liver and kidney were 48 HU and 79 HU, respectively. Additionally, for the proton therapy for lung cancer, an increased CT number (110–250 HU) due to contrast agent in the heart and great vessels was reported to cause a range error of about 1.0 cm [22]. We observed similar CT number differences between CE-pCT and VNC-pCT in the heart (230 HU) and great vessels (208 HU). In future work, a plan comparison study between CE-pCT and VNC-pCT for proton and heavy ion therapy is required.

### Limitation

The results of VNC generation showed not only a decrease in CT numbers in contrast regions but also in bone regions. The cause of this phenomenon can be traced to the dataset used for model training, where a comparison between the CE CT and VNC images revealed a disparity in bone CT numbers. The VNC CT images displayed lower bone CT numbers than the CE CT images, reflecting an inherent dataset tendency. As a result, the model’s predicted images also showed reduced bone CT numbers. This issue should be addressed in future research to ensure alignment between the bone CT numbers in the generated VNC-Net images and those in the input contrast-enhanced images (or TNC CT images). Methods such as segmenting the bone and adding it later via a segmentation task, which is one of the highest performing fields in medical artificial intelligence, or using a translation model that utilizes anatomical structures, can be explored.

The patient cohort and number of patients in this study were not optimal. We designed a retrospective study and searched for cases where patients were scanned with both CE-pCT and TNC-pCT. However, our institution typically acquires only one type of CT scan (either CE or TNC), leading to a limited sample size. When we can include more patients, we plan to categorize liver cancer cases based on the location of liver cancer (i.e., beam pathlength through the CE regions). Our current study includes only ten patients, which limits the ability to establish a significant difference in CT numbers. For the liver patients, the beam passed through the contrast-enhanced regions in the liver and right kidney. However, since we did not analyze beam weight for each angle, we were unable to determine how much beam weight passing through contrast-enhanced regions influenced the dose delivered to the PTVs. When the PTV is near the kidneys or heart, the dose delivered might be affected by changes in CT numbers.

## Conclusion

This study demonstrated that a deep learning framework can effectively translate from CE CT images into VNC images. The proposed model, VNC-Net, successfully generated VNC images within a DECT system and generalized its translation ability to planning CT scans through a deterministic image conversion between the planning CT and DECT. Through comprehensive analysis and transformation processes, our study highlights the potential of deep learning models in enhancing RT planning and imaging accuracy. However, despite the overall good performance of VNC-Net, it exhibited a limitation: the decrease in CT numbers in both contrast and bone regions, which was due to the dataset characteristics. Future work should focus on maintaining the CT numbers of bone, which are unrelated to the contrast agent.
